# The genetics of ray pattern variation in *Caenorhabditis briggsae*

**DOI:** 10.1186/1471-2148-5-3

**Published:** 2005-01-05

**Authors:** Scott Everet Baird, Cynthia R Davidson, Justin C Bohrer

**Affiliations:** 1 Department of Biological Sciences, Wright State University, Wright State University, Dayton OH 45435, USA; 2 Department of Pulmonary Medicine, Children's Hospital Medical Center, Cincinnatti OH 45229-3039, USA; 3 College of Medicine, Cleveland Clinic Foundation NA24, 9500 Euclid Avenue, Cleveland, OH 44195, USA

## Abstract

**Background:**

How does intraspecific variation relate to macroevolutionary change in morphology? This question can be addressed in species in which derived characters are present but not fixed. In rhabditid nematodes, the arrangement of the nine bilateral pairs of peripheral sense organs (rays) in tails of males is often the most highly divergent character between species. The development of ray pattern involves inputs from hometic gene expression patterns, TGFβ signalling, Wnt signalling, and other genetic pathways. In *Caenorhabditis briggsae*, strain-specific variation in ray pattern has provided an entrée into the evolution of ray pattern. Some strains were fixed for a derived pattern. Other strains were more plastic and exhibited derived and ancestral patterns at equal frequencies.

**Results:**

Recombinant inbred lines (RILs) constructed from crosses between the variant *C. briggsae *AF16 and HK104 strains exhibited a wide range of phenotypes including some that were more extreme than either parental strain. Transgressive segregation was significantly associated with allelic variation in the *C. briggsae *homolog of *abdominal B*, *Cb-egl-5*. At least two genes that affected different elements of ray pattern, ray position and ray fusion, were linked to a second gene, *mip-1*. Consistent with this, the segregation of ray position and ray fusion phenotypes were only partially correlated in the RILs.

**Conclusions:**

The evolution of ray pattern has involved allelic variation at multiple loci. Some of these loci impact the specification of ray identities and simultaneously affect multiple ray pattern elements. Others impact individual characters and are not constrained by covariance with other ray pattern elements. Among the genetic pathways that may be involved in ray pattern evolution is specification of anteroposterior positional information by homeotic genes.

## Background

A fundamental problem of morphological evolution is how does microevolution relate to macroevolution [1]. Are variation and selection within species sufficient to account for genetic divergence between species? One approach to this problem has been comparative developmental genetic studies in closely related species. This approach is complicated by developmental redundancy and cryptic variation [2-4]. Developmental redundancy occurs in species pairs in which similar adult morphologies are attained through distinct developmental processes. For example, in the nematodes *Caenorhabditis elegans *and *Oscheius tipulae*, cells in the vulval equivalence group that do not participate in vulval development are eliminated through fusion with the multinucleated syncytial epidermis [5-8]. In *Pristionchus pacificus*, that same outcome is achieved through apoptosis [9]. Cryptic variation occurs in species pairs in which the same developmental process is regulated through divergent genetic mechanisms. For example, *Drosophila melanogaster *and *D. simulans *have similar numbers and patterns of bristles. Hybrids have fewer bristles than either parental species [10,11]. Thus, the conserved bristle phenotypes of *D. melanogaster *and *D. simulans *mask cryptic variation in the genetic regulation of bristle pattern.

Another approach has been the study of morphological variation within species. In the butterfly *Bicyclus anynana*, expression patterns of *distalless *(*dll*), *engrailed *(*en*), and *spalt *(*sal*) coincide with eyespot coloration patterns [12,13]. Moreover, allelic variation in *distalless *has been associated with size variation in eyespots [12]. Thus, evolution of eyespot patterns may have resulted from selection for variant alleles of *dll*, *en*, and *sal*. Consistent with this model, conservation of the expression patterns of these genes in developing eyespots has been demonstrated in multiple butterfly species [13]. Developmental variation also has been characterized in several nematode species. For example, variant patterns of cell division in the ventral epidermis have been described in *C. elegans*, *C. briggsae*, and *O. tipulae *[14].

Another promising model for microevolutionary studies of morphological evolution is ray pattern variation in rhabditid nematodes. The rays are male-specific peripheral sense organs that mediate mating behavior [15-17]. In most rhabditid species there are nine bilateral pairs of rays embedded within the copulatory bursa of the tail [17]. The pattern of rays; their anteroposterior placement and the dorsoventral position of their sensory endings, generally are constant within species but often are the most divergent character between species [18]. Ray pattern in adult males is determined by cell contacts that form between ray structural cells and the surrounding cells of the lateral epidermis in L4 larvae [19,20]. These contacts in turn are determined by the specification of 'ray identities' [21]. In *Caenorhabditis elegans*, ray identities are specified through the integration of several regulatory inputs including: positional information from homeotic genes [22-26]; TGF-beta-like signalling [27-29]; Wnt signalling [25]; and ephrin/semaphorin signalling [30].

The ray pattern of *Caenorhabditis briggsae *differs from that of *C. elegans *in the placement of ray 3 [31,32]. In *C. elegans *males, ray 3 is located at the cloaca and is separate from from all other rays. In the canonical *C. briggsae *ray pattern, ray 3 is located in a posterior cluster of rays 3–6 and frequently is fused with ray 4. The *C. elegans *ray pattern is shared with several other *Caenorhabditis *species and is ancestral to the Elegans-group [33], a monophyletic clade that includes *C. briggsae *[34-36]. The *C. briggsae *ray pattern is mimicked by several *C. elegans *mutations including mutations in homeotic genes or in genes that regulate homeotic gene expression patterns [21,22,26,37,38]. Thus, the derived *C. briggsae *ray pattern may have arisen through changes in the specification of anteroposterior positional identities. It now is possible to address this hypothesis as variant *C. briggsae *strains that express an ancestral ray pattern at high frequencies have been identified [39]. In this paper, the segregation of ray pattern phenotypes in crosses between *C. briggsae *strains is described.

## Results

### The elements of ray pattern

**Figure 1:**
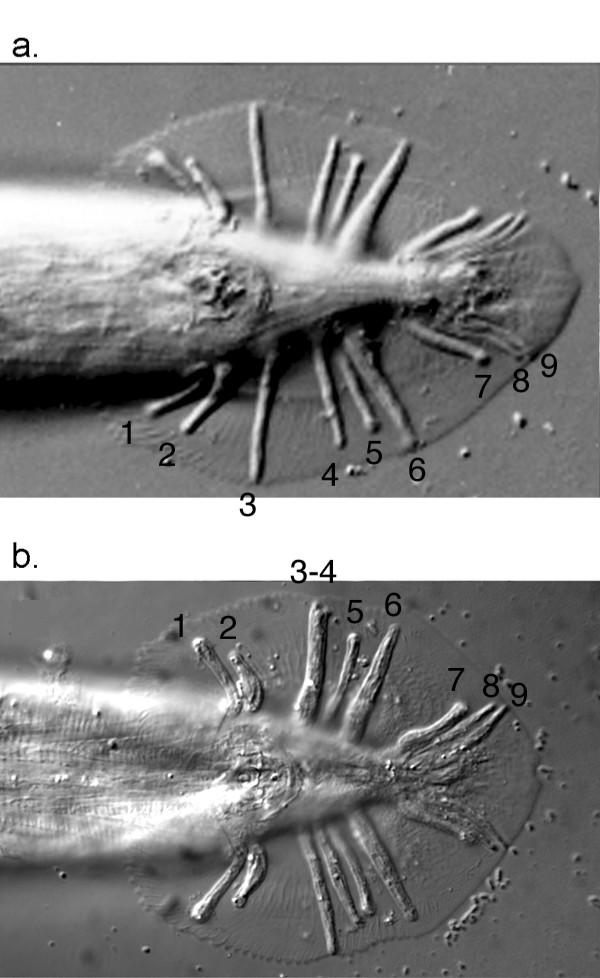
Ray patterns of *C. briggsae *and *C. elegans*. Ventral views of a) *C. elegans *and b) *C. briggsae *male tails. Anterior to the left. Left side up. Bilateral pairs of rays are numbered from anterior to posterior. a) *C. elegans *pattern in which ray 3 is separate from all other rays. This pattern is referred as a 2(1)3+3 pattern. b) *C. briggsae *pattern in which ray 3 is clustered with rays 4 – 6. This pattern is referred to as a 2/4+3 pattern. In this pattern ray 3 may be either free (right side) or fused with ray 4 (left side). The 2(1)3+3 pattern is ancestral to the Elegans-group, a monophyletic clade that includes *C. elegans *and *C. briggsae *[34].

The male-specific rays of rhabditid nematodes are embedded within a lateral fold of cuticle called the bursa or tail fan [19] (Figure 1). The sensory endings of most rays are attached to and open through the surface of the bursa. Ray pattern refers collectively to the anteroposterior positions of each ray, the surface (dorsal, ventral or medial) through which their sensory endings are exposed, and whether or not individual rays are fused with each other. Rays also differ in regard to neurotransmitter usage [5,21,40]. Mutations that affect ray pattern also affect neurotransmitter usage indicating that multiple properties of rays arise through the specification of 'ray identities' [21,24].

The derived ray pattern of *C. briggsae *entails the posterior displacement of ray 3 from a position level with the cloaca to the post-cloacal cluster of rays 4 – 6 and the frequent fusion of ray 3 with ray 4 [31,32,34]. This ray pattern is exhibited in nearly all males of *C. briggsae *strains AF16 and VT847 [39]. Ray pattern in *C. briggsae *strains HK104, HK105, and PB800 is more variable. These strains exhibit the derived and ancestral ray patterns at approximately equal frequencies [39].

### Segregation of *C. briggsae *ray pattern phenotypes

**Figure 2:**
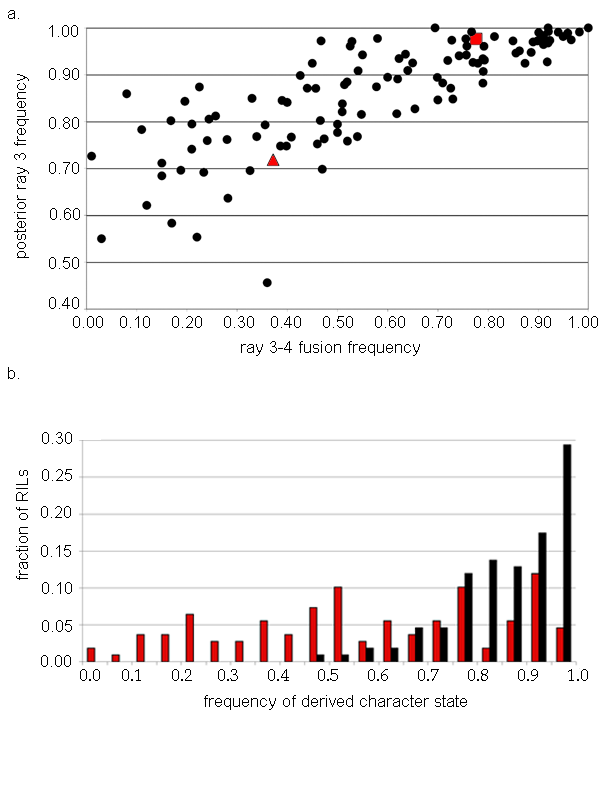
Segregation of ray pattern phenotypes in *C. briggsae *RILs. a) Scatter plot comparing frequencies of derived character states for the position of ray 3 (Y-axis) and fusion of rays 3 and 4 (X-axis) in RILs. Each point represents an individual strain for which a minimum of 100 sides were scored for ray pattern. Parental strains, AF16 and HK104 represented by red square and red diamond, respectively. RILs represented by closed circles. Correlation coefficient, r, for ray position and fusion equaled 0.80. b) Frequency distributions for derived character states of ray 3 position (black bars) and ray 3–4 fusion (red bars) among RILs.

The genetic basis of ray pattern variation in *C. briggsae *was characterized through the segregation of ray pattern phenotypes in a set of recombinant inbred lines (RILs). These RILs were constructed from a cross between strains AF16 and HK104. Individual RILs were established from F2 progeny of this cross. RILs were inbred, one hermaphrodite per generation, through F11. Based on this degree of inbreeding, 99.9% of loci in each RIL should be homozygous. RILs were scored for two aspects of ray pattern; the position of ray 3 and the fusion of rays 3 and 4 (Figure 2).

Several RILs exhibited ancestral or derived phenotypes at frequencies more extreme than either parental strain (Figure 2a). This was true for both the position of ray 3 and for fusions of rays 3 and 4. For ray 3 position, one quarter of the RILs exhibited phenotypes more extreme than either parental strain. For ray fusions, half of the RILs were more extreme than the parental strains. Such transgressive segregation is best explained through the presence, in both AF16 and HK104, of alleles at some loci that promoted ancestral character states and alleles at other loci that promoted derived character states. The high frequency of RILs with extreme phenotypes indicated that both parental strains likely were fixed for antagonistic alleles at multiple loci.

A relatively strong correlation was observed between variation in the position of ray 3 and its fusion with ray 4 (r = 0.797; Figure 2a). This correlation was highly significant (p = 2.5 × 10^-23^). This was expected as rays 3 and 4 could not have fused unless they were adjacent to each other [19] and because the placement of rays and fusion between rays have been considered to be pleiotropic phenotypes derivative of the specification of ray identities [19,22-25,27-30]. If anything, it was surprising that the correlation was not stronger. Based on the observed correlation coefficient, approximately one third of the variation in ray fusion frequency was independent of variation in the position of ray 3. This was readily apparent when the frequency distributions of these two ray pattern elements were compared (Figure 2b). The frequency distribution for the placement of ray 3 was highly skewed toward the derived phenotype. The frequency distribution for fusions of rays 3 and 4 was much broader and was only slightly skewed. The differences in the shapes of these distributions were significant (Kolmgorov-Smirnov, D = 0.4396, p < 0.001).

Neither the frequency distribution for the placement of ray 3 nor that for the fusion of ray3 to ray 4 were normal (p = 0.00 and p = 0.03, respectively; Figure 2b). The frequency distribution for the placement of ray 3 was highly skewed toward the derived phenotype. This result was inconsistent with the placement of ray 3 being governed strictly by additive affects. More likely, the placement of ray 3 was regulated by epistasis. Twentynine per cent of RILs exhibited the most extreme phenotype (Figure 2b). Fixation of derived alleles at as few as two major-effect genes could have been sufficient to ensure a posterior localization of ray 3 at this frequency. The frequency distribution for fusions of rays 3 and 4 was very broad and and only slightly skewed. This was consistent with ray fusion being a complex character with inputs from multiple developmental processes. At least one of these processes was shared with ray positioning; rays 3 and 4 could not have fused if they were not adjacent.

### Association of ray pattern with allelic variation in homeotic genes

**Figure 3:**
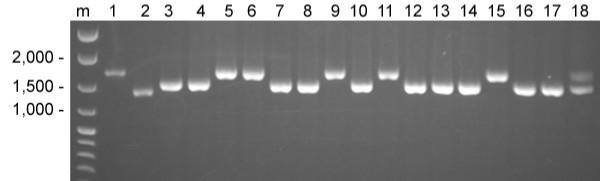
Allelic variation of *Cb-egl-5*. *Cb-egl-5 *amplification products of 1) AF16; 2) PB800; 3) HK104; 4–17) selected RILs; 18) an HK104/AF16 heterozygote. The expected amplification product size based on the *C. briggsae *AF16 genome sequence was 1,723 bp. m) Marker DNA, sizes of selected markers as indicated.

The derived *C. briggsae *ray pattern may have evolved through changes in homeotic gene expression patterns [33,39]. The homeotic genes most likely to affect ray pattern are *mab-5 *and *egl-5*. These genes are expressed in the posterior body and tail regions of *C. elegans *[40] and mutations in them can alter ray pattern [22]. Allelic variants that affect the size of the first intron of *Cb-egl-5 *have been identified (Figure 3). The AF16::HK104 RILs were genotyped for these variants to test *Cb-egl-5 *for association with ray pattern variation [see Additional File 1].

Significant associations were observed between *Cb-egl-5 *allelic variants and both the position of ray 3 and the fusion of ray 3 to ray 4 (Table 1). These associations were transgressive; the HK104 allele of *Cb-egl-5 *cosegregated with derived phenotypes, the AF16 allele with ancestral phenotypes. Allelic variants in *Cb-mab-5 *have not been identified. However, the *Cb-mab-5 *(=CBG00029) and *Cb-egl-5 *(=CBG00023) are located within 60 kb of each other [41,42]. Because of this close physical linkage, allelic variants in these genes very likely cosegregated. Thus, allelic variation in *Cb-mab-5*, *Cb-egl-5*, and/or other linked loci may have been responsible for observed transgressive associations.

**Table 1:** Cosegregation of *Cb-egl-5 *with ray pattern phenotypes.

	allele^1^		
phenotype	AF16	HK104	p value
ray position	0.81	0.90	6.2 × 10^-4^
ray fusion	0.42	0.68	4.2 × 10^-5^

^1 ^Mean frequency of derived character states exhibited in RILs homozygous for either parental *Cb-egl-5 *allele.

### *mip-1 *marker-assisted introgression

The segregation of phenotypes in the RILs indicated that multiple major-effect and additive loci were involved in ray pattern variation in *C. briggsae*. Because a high density recombination map for *C. briggsae *has not yet been constructed, it is not possible to identify these loci through QTL analyses. However, a limited number of mutations with visible phenotypes have been mapped to *C. briggsae *chromosomes [B. Gupta, pers. comm., [43]]. These mutations all were generated in an AF16 background. One such mutation defines the gene *mip-1*. *mip-1 *very likely is the *C. briggsae *homolog of *C. elegans unc-22 *[D. Baillie, pers. comm.]. In *C. elegans*, *unc-22 *is located on chromosome IV and is linked to several genes known to regulate homeotic gene expression patterns, e.g. *lin-49 *which encodes a bromodomain protein thought to be involved in chromatin remodeling [26]. *mip-1 *marker-assisted introgression was used to determine if any genes linked to it were involved in ray pattern variation in *C. briggsae*.

**Figure 4:**
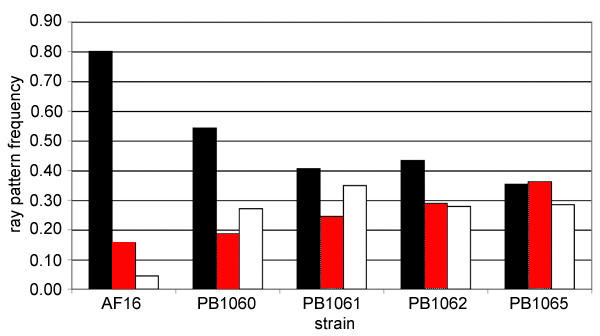
Comparisons of ray pattern phenotypes in *mip-1 *introgressed strains. Frequencies of ray pattern phenotypes exhibited in AF16 and four *mip-1 *introgressed strains. Black bars represents ray 3 in a posterior position and fused with ray 4. Red bars represents ray 3 in a posterior position but not fused with ray 4. White bars represents ray 3 in an anterior postion.

Four *C. briggsae *strains were constructed in which HK104 DNA, in the region of *mip-1*, has been introgressed into an otherwise AF16 background. This was accomplished through a series of backcrosses that were initiated by mating HK104 males to *mip-1 *(AF16) hermaphrodites. For each introgressed strain, F1 through F6 males were crossed to *mip-1 *(AF16) hermaphrodites. Wild-type F7 hermaphrodites were selected and propagated by self-fertilization. From each F7 hermaphrodite, multiple wild-type F8 hermaphrodites were picked. Homozygous strains were established from F8 hermaphrodites that segregated only wild-type progeny. From each set of backcrosses, a single introgressed strain was retained. These introgressed strains were scored for the position of ray 3 and for ray 3–4 fusions (Figure 4). The ray patterns of all four introgressed strains differed significantly from AF16 both in the placement of ray 3 and in the frequency with which ray 3 fused to ray 4 (Table 2). Significant differences also were apparent between some pairs of introgressed strains, most notably between PB1060 and PB1065. PB1060 and PB1065 did not differ in the placement of ray 3 but only in the frequency with which ray 3 fused to ray 4 (Figure 4; Table 2). Thus, it appears that allelic variation in at least two genes linked to *mip-1 *is involved in ray pattern variation. One of these genes affects the position of ray 3 and possibly the frequency of ray fusion. The HK104 allele of this gene was present in all *mip-1 *introgressed strains. The other gene affects only the frequency of ray fusion. The HK104 allele of this gene was present in PB1065 but not in PB1060. As more *C. briggsae *mutant strains become available, it should be possible to identify these genes through additional introgression studies coupled with genotypic characterizations of introgressed DNA.

**Table 2:** Comparison of *mip-1 *introgressed lines.

	p values for reciprocal chi-squared tests^1^				
	AF16	PB1060	PB1061	PB1062	PB1065
ray pattern^2^					
AF16	---	9.6 × 10^-30^	3.3 × 10^-54^	6.5 × 10^-32^	5.3 × 10^-46^
PB1060	7.9 × 10^-4^	---	0.031	0.093	1.2 × 10^-5^
PB1061	2.2 × 10^-7^	0.026	---	0.367	0.011
PB1062	2.8 × 10^-6^	0.092	0.293	---	0.091
PB1065	2.3 × 10^-9^	1.3 × 10^-4^	0.038	0.151	---
ray position^3^					
AF16	---	6.9 × 10^-4^	1.5 × 10^-5^	3.6 × 10^-4^	3.2 × 10^-4^
PB1060	1.32 × 10^-29^	---	0.068	0.72	0.67
PB1061	6.4 × 10^-53^	0.053	---	0.12	0.14
PB1062	8.3 × 10^-30^	0.73	0.16	---	0.95
PB1065	1.65 × 10^-36^	0.66	0.14	0.95	---
ray fusion^4^					
AF16	---	0.13	5.3 × 10^-3^	3.1 × 10^-3^	9.8 × 10^-6^
PB1060	0.013	---	0.061	0.033	3.1 × 10^-5^
PB1061	4.74 × 10^-6^	0.057	---	0.80	0.029
PB1062	9.37 × 10^-7^	0.030	0.80	---	0.052
PB1065	1.33 × 10^-16^	1.59 × 10^-6^	0.013	0.028	---

^1 ^Probabilities in each column were determined using frequencies of different ray patterns in each strain (column head) as the null hypothesis.

^2 ^p values for the complete ray pattern (3 posterior and fused, 3 posterior not fused, 3 anterior).

^3 ^p values for ray placement (anterior vs posterior).

^4 ^p values for fusion of rays 3 and 4 when ray 3 is in a posterior position.

## Discussion

Ray pattern in *C. briggsae *varies with respect to the placement of ray 3 and fusions of ray 3 to ray 4. Ancestral states for these characters in the Elegans-group of *Caenorhabditis *are an anterior location, level with the cloaca, and the absence of ray fusions [34]. Derived states are a posterior location, clustered with rays 4 through 6, and fusion of rays 3 and 4. An unexpected result was the incomplete correlation between the position or ray 3 and its fusion with ray 4. In *C. elegans*, mutations that alter ray position have been accompanied by ray fusion. For this reason, ray position and ray fusion were thought to be pleiotropic phenotypes that arose from the specification of ray identities [19,22-25,30]. Because of this, it was expected that ray pattern evolution would involve changes in suites of covariant characters rather than independent modification of individual ray pattern elements. However, only two-thirds of variation in ray fusion resulted from variation in ray position (r^2 ^= 0.635). Moreover, evidence for a gene affecting only ray fusion was obtained from comparisons of ray patterns exhibited by the *mip-1 *introgressed strains. Hence, variation in ray pattern evolution is not wholey constrained by the specification of ray identities and other ray pattern elements, such as neurotransmitter usage, also may vary independently. A similar pattern of partial constraint and flexibility has been observed for variation in eyespots size in the butterfly *Bicyclus anynana *[12].

Two haplotypes of *C. briggsae *have been described [44] and variation in ray pattern follows haplotype structure [39]. Strains in haplotype 1, including AF16, exhibit almost exclusively a derived ray pattern. Strains in haplotype 2, including HK104, exhibit derived and ancestral ray patterns at equal frequencies. One interpretation of these results is that full expression of the derived ray pattern never became fixed within haplotype 2. However, a *C. briggsae*-like ray pattern also has been reported for *C. clavopapillata *[45], another species within the Elegans-group. *C. clavopapillata *has not been observed since its first description and some authors have considered it and *C. briggsae *to be synonymous [34]. If *C. clavopapillata *is distinct from *C. briggsae*, the posterior position of ray 3 and its frequent fusion with ray 4 may be ancestral for these two species. This would make the *C. elegans*-like ray pattern in haplotype 2 an atavistic character. It should be possible to discriminate between these two models either through the characterization of additional *C. briggsae *strains or through the redescription and molecular characterization of *C. clavopapillata*.

Regardless of their evolutionary history, haplotypes 1 and 2 provided an entrée to the genetics of morphological variation of ray pattern in *C. briggsae*. The relatively slight difference between the AF16 and HK104 phenotypes hid a wealth of genetic variation. This was evident in the nearly continuous variation exhibited in RILs for both the position of ray 3 and its fusion with ray 4. This variation included many RILs with phenotypes more extreme than either parental strain. Such transgressive segregation is common in both plants and animals and is thought to arise through fluctuating selection and/or through stabilizing selection at minor QTL when directional selection at major QTL overshoots a phenotypic optimum [46]. The transgressive segregation of ray pattern phenotypes may have resulted from selection on homeotic gene expression patterns. In *C. elegans*, ray 3 precursor cells are born at the junction of the *mab-5 *and *egl-5 *expression domains [23,47]. These homeotic genes are required for the specification of positional identities in the posterior body and tail regions, respectively [47,48] and some mutations in them cause the *C. elegans *ray pattern to phenocopy that of *C. briggsae *[22,26]. We have demonstrated a significant transgressive association between allelic variation at *Cb-egl-5 *and variation in ray pattern. As *Cb-egl-5 *and *Cb-mab-5 *are closely linked, variation in either or both of these genes may be responsible for the observed association. Alternatively, linkage to *Cb-egl-5 *may be coincidental, and ray pattern variation in *C. briggsae *may not result from variation in homeotic gene expression patterns. The best test of these alternative models will be the high resolution mapping of the allelic variants responsible for ray pattern variation.

Loci not linked to the homeotic gene cluster also must be involved in ray pattern variation. Direct evidence for this was obtained from the *mip-1 *marker assisted introgression studies. The *C. elegans *homolog of *mip-1*, *unc-22*, is not linked to the the homeotic gene cluster and linkage of *mip-1 *to *Cb-egl-5 *and *Cb-mab-5 *is unlikely. There appear to be at least two loci that affect ray pattern linked to *mip-1*. One of these affected both the placement of ray 3 and its fusion with ray 4. The other affected only ray fusion. These associations were not transgressive, i.e. the allelic variants linked to *mip-1 *antagonized the allelic variants linked to *Cb-egl-5*. If the transgressive segregation linked to *Cb-egl-5 *does result from allelic variation in one or more homeotic genes, then the antagonistic variation linked to *mip-1 *may be in genes that regulate homeotic gene expression patterns. A direct test of this hypothesis is possible. Several genes required for proper regulation of homeotic gene expression patterns have been identified in *C. elegans *[25,26,37,38,49]. *C. briggsae *homologs of these genes could be tested for association with ray pattern variation in the RILs. Ideally, these tests would be integrated into a genome wide screen for variant loci with effects on ray pattern. This will require the enhancement of genetic resources available for *C. briggsae*.

## Conclusions

Ray pattern in *Caenorhabditis *provides a powerful model for the study of morphological evolution. Macroevolutionary comparisons between species and microevolutionary analyses of variation within species are possible. Augmenting these approaches is a detailed understanding of the genetic and cellular basis of ray pattern development in *C. elegans*. In *C. briggsae*, intraspecific variants have been characterized that affect the expression of ancestral and derived ray patterns. These variants have a complex genetic basis involving multiple genes. Some of these genes exhibit transgenic segregation, some affect all of elements of ray pattern, and some that affect only a subset of ray pattern elements. At least one gene that affects ray pattern variation in *C. briggsae *is linked to the homeotic gene cluster. Thus, ray pattern variation may result from altered expression patterns of homeotic genes. Further characterizations of the genetics of ray pattern variation will test this model and will address interactions between different genes that impact ray pattern in *C. briggsae*.

## Methods

### Strains and strain maintenance

*C. briggsae *strains AF16, and HK104 are available from the *Caenorhabditis *Genetics Center [50]. These strains were maintained on agar plates seeded with *E. coli *strain OP50. Recombinant inbred lines (RIL) were constructed starting with HK104 males mated to sperm-depleted AF16 hermaphrodites [15]. Each RIL was initiated with a single F2 hermaphrodite and propagated through F11, one hermaphrodite per generation.

### Microscopy

Ray pattern phenotypes were scored using differential interference contrast optics at a magnification of 400× [5]. Right and left sides were scored independently [39]. Microscopic images of worms anethesized in 0.2% sodium azide were digitally captured using a Spot Camera and Software (Diagnostic Instruments, Inc., Sterling Heights MI).

### PCR amplification

Forward and reverse primers for PCR amplifications *Cb-egl-5*, CAGGGAGCGGACAACTTCAAAGG and GGACACAGCCCAGGATTAGCGAC, respectively, were designed based on genome sequence data from *C. briggsae *strain AF16 [41]. Amplification products were size fractionated by electrophoresis through 1% agarose.

### Statistical analyses

Pearson's product moment correlation cofficients between ray pattern elements in RILs and Wilcoxon nonparametric tests of the association between *Cb-egl-5 *allelic variation with ray pattern variation were determined online at [51]. Frequency distributions of the segregation of ray 3 positional and fusion phenotypes were compared using the Kolmogorov-Smirnov tests as implemented at [52]. Ray pattern phenotypes of *mip-1 *introgressed strains were compared using reciprocal chi-squared tests using Excel v10.1.0 (Microsoft, Inc., Redman WA).

## Abbreviations

RIL = recombinant inbred line; QTL = quantitative trait loci; *dll *= *distalless*; *en *= *engrailed*; *sal *= *spalt*

## Author's contributions

**SEB**: Planned and supervised all research activities. Participated in construction and genotyping of RILs. Reviewed all statistical analyses. Wrote manuscript.

**CRD**: Constructed, genotyped, and characterized ray pattern phenotypes of RILs. Statistical analyses of the segregation of ray pattern phenotypes and association of *Cb-egl-5 *variation with ray pattern variation. Reviewed manuscript.

**JCB**: Constructed and characterized phenotypes of *mip-1*-assisted introgressed lines. Statistical analyses of ray pattern phenotypes of *mip-1 *introgressed lines. Reviewed manuscript.

## Supplementary Material

Additional File 1 AF16 × HK104 RIL ray pattern data. *Cb-egl-5 *genotypes and ray pattern phenotypes of RIL derived from AF16 × HK104. Click here for file
